# Optimized Conformal Total Body Irradiation Among Recipients of TCRαβ/CD19-Depleted Grafts in Pediatric Patients With Hematologic Malignancies: Single-Center Experience

**DOI:** 10.3389/fonc.2021.785916

**Published:** 2021-12-16

**Authors:** Daria Kobyzeva, Larisa Shelikhova, Anna Loginova, Francheska Kanestri, Diana Tovmasyan, Michael Maschan, Rimma Khismatullina, Mariya Ilushina, Dina Baidildina, Natalya Myakova, Alexey Nechesnyuk

**Affiliations:** ^1^ Department of Radiation Oncology, Dmitry Rogachev National Research Center of Pediatric Hematology, Oncology and Immunology, Moscow, Russia; ^2^ Department of Hematopoietic Cell Transplantation, Dmitry Rogachev National Research Center of Pediatric Hematology, Oncology and Immunology, Moscow, Russia; ^3^ Department of Pediatric Hematology and Oncology, Dmitry Rogachev National Research Center of Pediatric Hematology, Oncology and Immunology, Moscow, Russia; ^4^ Department of Onco-hematology, Dmitry Rogachev National Research Center of Pediatric Hematology, Oncology and Immunology, Moscow, Russia

**Keywords:** TBI, IMRT, total body irradiation, Total marrow and lymphoid irradiation, acute leukemia, pediatric patients, boost to bone marrow, TomoTherapy

## Abstract

Total body irradiation (TBI) in combination with chemotherapy is widely used as a conditioning regimen in pediatric and adult hematopoietic stem cell transplantation (HSCT). The combination of TBI with chemotherapy has demonstrated superior survival outcomes in patients with acute lymphoblastic and myeloid leukemia when compared with conditioning regimens based only on chemotherapy. The clinical application of intensity-modulated radiation therapy (IMRT)-based methods (volumetric modulated arc therapy (VMAT) and TomoTherapy) seems to be promising and has been actively used worldwide. The optimized conformal total body irradiation (OC-TBI) method described in this study provides selected dose reduction for organs at risk with respect to the most significant toxicity (lungs, kidneys, lenses). This study included 220 pediatric patients who received OC-TBI with subsequent chemotherapy and allogenic HSCT with TCRαβ/CD19 depletion. A group of 151 patients received OC-TBI using TomoTherapy, and 40 patients received OC-TBI using the Elekta Synergy™ linac with an Agility-MLC (Elekta, Crawley, UK) using volumetric modulated arc therapy (VMAT). Twenty-nine patients received OC-TBI with supplemental simultaneous boost to bone marrow—(SIB to BM) up to 15 Gy: 28 patients (pts)—TomoTherapy; one patient—VMAT. The follow-up duration ranged from 0.3 to 6.4 years (median follow-up, 2.8 years). Overall survival (OS) for all the patients was 63% (95% CI: 56–70), and event-free survival (EFS) was 58% (95% CI: 51–65). The cumulative incidence of transplant-related mortality (TRM) was 10.7% (95% CI: 2.2–16) for all patients. The incidence of early TRM (<100 days) was 5.0% (95% CI: 1.5–8.9), and that of late TRM (>100 days) was 5.7 (95% CI: 1.7–10.2). The main causes of death for all the patients were relapse and infection. The concept of OC-TBI using IMRT VMAT and helical treatment delivery on a TomoTherapy treatment unit provides maximum control of the dose distribution in extended targets with simultaneous dose reduction for organs at risk. This method demonstrated a low incidence of severe side effects after radiation therapy and predictable treatment effectiveness. Our initial experience demonstrates that OC-TBI appears to be a promising technique for the treatment of pediatric patients.

## Introduction

Total body irradiation (TBI) in combination with chemotherapy is widely used worldwide as a conditioning regimen prior to transplanting hematopoietic stem cells in patients with malignant hematological diseases.

The main benefits of TBI include tumor cell elimination and general immunosuppressive effects. The combination of TBI with chemotherapy has demonstrated superior survival results in patients with acute lymphoblastic and myeloid leukemia when compared with conditioning regimens, including chemotherapy alone (1–7). However, TBI-based regimens show significant disadvantages for intermediate and long-term toxicity, especially pulmonary toxicity (up to 33% incidence of grade 3+) (8, 9). The incidence of pneumonitis after TBI-conditioning regimens varies, covering a range of 10.3%–45%, and it depends on many factors, such as patient characteristics and treatment technique (10–12). For many years, conventional techniques using low-dose rates (5–15 cGy/min) and lung shielding have historically been a method of choice for TBI treatment (13–16). The irradiation of healthy organs and tissues with high radiosensitivity, such as the lungs and kidneys, may occasionally bring about lethal side effects (8, 11, 17). The disadvantage of the conventional TBI treatment technique is the lack of sparing organs at risk (OARs), with the exception of the lungs. In addition, there is no capability to measure the dose in a small voxel volume and to create dose/volume histograms (DVHs) for the planning target volume (PTV) and OARs to correlate toxicities with received radiation doses.

The clinical application of modern radiotherapy methods, such as intensity-modulated radiation therapy (IMRT), as well as the feasibility of irradiation of extended targets with helical TomoTherapy, have been investigated for TBI and total bone marrow and lymphoid irradiation (TMLI) in adult patients (18–21). However, the number of reports on the application of these methods for the treatment of pediatric patients remains limited (22–25).

In our center, pediatric patients with hematologic malignancies receive TCR-alpha/beta-depleted grafts to minimize the incidence of GVHD and to achieve fast immune reconstitution after HSCT (26–28).

Our goal was to develop and implement the optimized conformal total body irradiation (OC-TBI) method in pediatric practice as a part of the patient conditioning protocol prior to allogeneic bone marrow transplantation (29, 30). The OC-TBI method described in this study provides reproducible dose reduction for OARs that are prone to significant radiotoxicity (lungs, kidneys, lenses).

The main objective of OC-TBI is to irradiate the PTV with maximum homogeneity with simultaneous dose reduction to the critical organs with planning and treatment optimization based on the age of pediatric patients.

We present initial experience and results of implementing the new TomoTherapy- and VMAT-based OC-TBI method in pediatric practice and the toxicity and survival rates in TCRαβ/CD19-depleted graft recipients.

The IMRT-based OC-TBI method provides the opportunity to deliver additional doses to sanctuary sites (i.e., bone marrow, extramedullary sites) to improve radiation treatment effectiveness for advanced patients (30, 31).

We also report outcomes for patients with refractory leukemia who received local dose escalation (boost) to bone marrow.

## Materials and Methods

### Patient Characteristics

Two hundred and twenty (220) patients underwent optimized conformal IMRT-based total body irradiation (OC-TBI) in a myeloablative conditioning regimen at the Radiotherapy Department of the Dmitriy Rogachev National Medical Research Center of Pediatric Hematology, Oncology and Immunology in Moscow, Russia between July 2012 and September 2020. Radiation therapy was followed by chemotherapy and allogenic HSCT with TCRαβ/CD19 depletion. The majority of the patients had ALL (*n* = 165). All patients were included in the respective high-risk groups. Detailed patient characteristics are presented in **Table 1**. All the data were retrieved from the patients’ medical records.

**Table 1:** Patient and treatment characteristics.

Patients	(*n* = 220)
**Sex**	
Male	151 (69%)
Female	69 (31%)
Median (range) age at TBI (year)	10.2 (3.0–21.0)
**Disease**	
ALL	165 (75%)
*T-cell ALL*	*63 (38% of ALL)*
*B-cell ALL*	*102 (62% of ALL)*
AML	25 (11%)
Other (NHL, biphenotypic/bilineal leukemia, JMML)	30 (14%)
**Disease status at transplantation**	
ALL	
CR 1	44 (27%)
CR 2	86 (52%)
≥CR 3	23 (14%)
Active disease	12 (7%)
AML	
CR 2/3	5 (20%)
Active disease	20 (80%)
Others	
CR 1	9 (30%)
CR 2/3	14 (47%)
Active disease	7 (23%)
**Conditioning regimens**	
OC-TBI 12 Gy	220 (100%)
*SIB to BM up to 15 Gy*	*29 (13%)*
Fludarabine	220 (100%)
Thiotepa	164 (74%)
VP-16	49 (22%)
**Donor characteristics**	
Type of donor	
Haplo-	192 (88%)
MSD	14 (6%)
MUD	14 (6%)
**Cell dose infused, median (range)**	
CD34^+^ cells × 106/kg	9.21 (0.9–15.64)
αβ^+^ T cells × 103/kg	35.6 (4.3–377.2)

ALL, acute lymphoblastic leukemia; AML, acute myeloid leukemia; NHL, non-Hodgkin lymphoma; JMML, juvenile myelomonocytic leukemia; CR, complete remission; OC-TBI, Optimized Conformal Total Body Irradiation; SIB to BM, simultaneous integrated boost to bone marrow; VP-16, etoposide; MSD, matched sibling donor; MUD, matched unrelated donor.

Immediately after OC-TBI treatment, the patients received chemotherapy according to the different schedules. Conditioning regimens, donor type, and graft composition details are provided in **Table 1**.

Twenty-nine patients (13%) (19 with active disease prior to HSCT) received radiation treatment with simultaneous integrated boost to bone marrow (SIB to BM) with doses up to 15 Gy followed by chemotherapy in accordance with an individual schedule based on unfavorable performance status and/or the etiology of the main disease.

The study was approved by the local ethics committee of the Dmitriy Rogachev National Medical Research Center for Pediatric Hematology, Oncology and Immunology, in accordance with the Helsinki Declaration, and patients and/or their legal guardians provided informed consent to participate in the study.

### Radiation Therapy Preparation

#### CT Simulation

Our Radiation Therapy Department is equipped with one TomoTherapy™, Accuray Inc. (Sunnyvale, CA, USA) and two Elekta Synergy™ treatment units with an Agility-MLC (Elekta) and one CT scanner—GE LightSpeed RT16.

**Figure 1:**
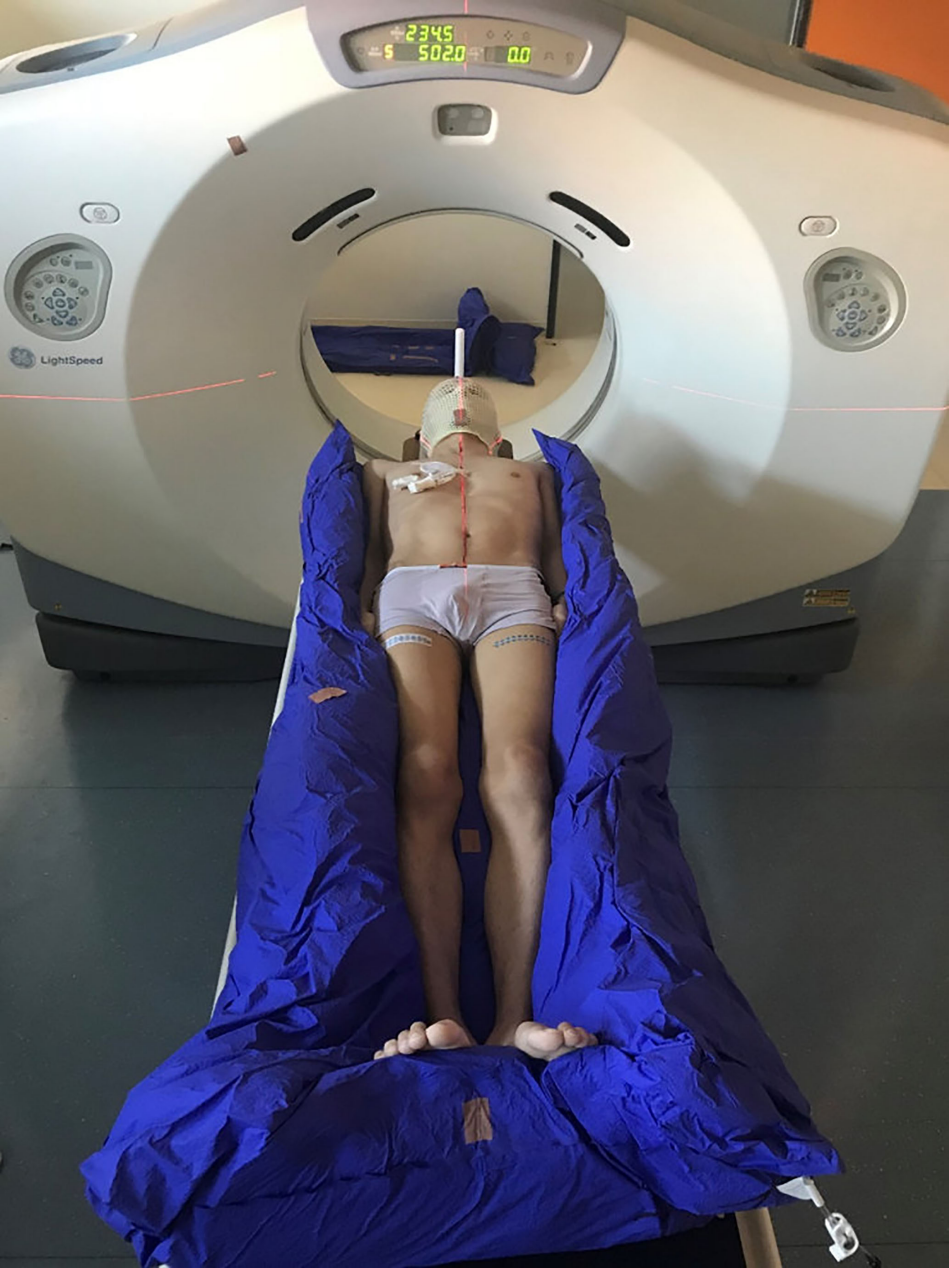
CT simulation using a vacuum bag for body and extremity fixation and a thermoplastic mask for head and neck fixation.

Approximately 1 week prior to the treatment, all the patients underwent CT simulation using individualized fixation. The patients were immobilized in the supine position, laid in vacuum bags for body and extremity fixation, and used thermoplastic masks for head and neck fixation, as shown in **Figure 1**.

Following patient fixation, planning CT images were acquired using slice thicknesses of 5 mm. Patients taller than 115 cm were scanned twice. The first scan included the upper part of the body down to the knee joints, and the second scan included legs from the toes up to the upper third of the thigh. A fiducial marker was placed in the middle of the thigh to assist in determining the juncture between the two images.

Thirty-two patients (15%) of younger ages underwent both CT simulation and radiotherapy treatment under general anesthesia.

#### Dose Prescription

Monaco 5.11 (Elekta Inc.) MIM Maestro™ software was used to contour the target and OAR volumes.

The lungs, kidneys, and lenses were selected as critical organs based on reported literature data (4, 6, 8–11, 32–35).

The PTV included the patient’s whole body minus critical structures (OARs). The following structures were created: external body contour, PTV (consisting of body without skin with 3 mm inner margin), eyes, lenses, lungs, and kidneys.

In cases when the patient’s body height exceeded 115 cm, the total body volume was divided into two planning target volumes—PTV_Body and PTV_Legs.

**Figure 2:**
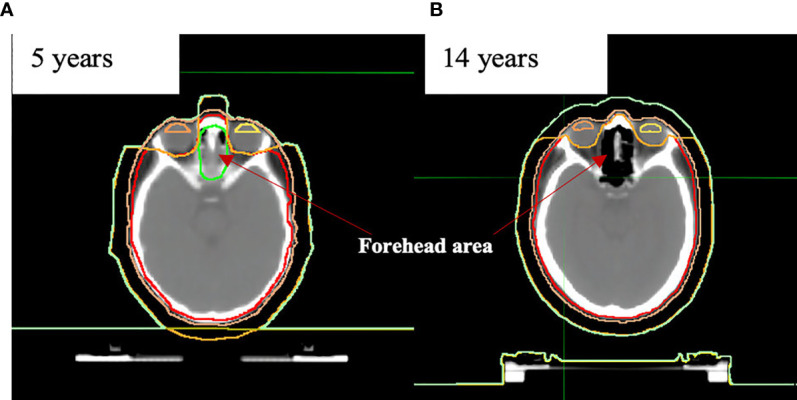
Anatomical differences in “forehead area” in the example of **(A)** 5- and **(B)** 14-year-old patients.

Additional contours were defined in support of treatment planning tasks. For dose control between eyes (small children have part of the brain located in this area), we additionally created the contour named the “forehead area” **(** **Figure 2**).

Ribs were contoured as an additional target volume within the PTV and with a set-prescribed Dmin for better control of steeply decreasing dose gradient in the area between lungs and PTV.

For the TomoTherapy patients, a virtual volume of 1 cm thickness was added to the PTV as an additional target (PTV+1 cm) and was used to account for the patient’s motion and breathing while providing the required dose to the skin (**Figure 2**). For the Elekta patients, we used the Monaco 5.11 (Elekta inc., UK, Crawley) Auto-Flash option with a 1-cm margin.

For the patients who received SIB to BM, the contoured addition structure PTV_1500 included all skeletal bones. Treatment volume for skeletal bones in the case of SIB was created without additional margin.

The prescribed total dose for the PTV was 12 Gy delivered in single fractions of 2.0 Gy or 3.0 Gy. The prescribed total doses for OAR are given in **Table 2**.

**Table 2:** Dose prescriptions with “target” and “acceptable” values.

Structure	Target value	Acceptable value
PTV	Mean dose (12 Gy) ± 2%	Mean dose (12 Gy) ± 5%
	D98% >11.4 Gy	D95% >11.4 Gy
	D2% <13 Gy	D5% <13 Gy
Forehead	D98% >11.4 Gy	D95% >11.4 Gy
	D2% <13 Gy	D5% <13 Gy
Ribs	D95% >10 Gy	D90% >10 Gy
Lungs	D99% >6 Gy	D90% > 6Gy
	V8 <40%	V8 <40%
Kidneys	Dmean <8 Gy	Dmean < 8Gy
Lenses	As low as achievable	

We established targeted and acceptable values for all the structures with the objective of optimizing treatment planning and plan optimization procedures (**Table 2**).

## Treatment Planning and Results

TomoTherapy 4.5 (Accuray Inc., Sunnyvale, CA, USA) and Monaco 5.11 (Elekta Inc.) planning systems were used for treatment planning. The received results with standard deviations after calculation of the treatment plans are presented in **Table 3**.

**Table 3:** Treatment plan calculation results.

Structure	TomoTherapy (*n* = 151)	VMAT Elekta (*n* = 40)
	Dose ± SD or %	Dose ± SD or %
PTV (Dmean)	12.05 ± 0.05	12.16 ± 0.12
Lung_L (Dmean)	7.88 ± 0.12	7.62 ± 0.14
Lung_R (Dmean)	7.84 ± 0.13	7.57 ± 0.14
Lung_L (V8)	37.55 ± 3.82	38.81 ± 2.79
Lung_R (V8)	36.52 ± 3.96	38.45 ± 2.66
Kidney_L (Dmean)	7.44 ± 0.42	7.31 ± 0.35
Kidney_R (Dmean)	7.49 ± 0.45	7.40 ± 0.29
Ribs (Dmean)	11.21 ± 0.12	11.62 ± 0.21
Lens_L	6.23 ± 0.55	6.13 ± 0.55
Lens_R	6.10 ± 0.75	6.30 ± 0.51
Forehead/Brain	12.01 ± 0.04	12.32 ± 0.09

The separate treatment plan was created for PTV_Legs in feet-first rotate position with the 5-cm “juncture area” between “body” and “legs” treatment volumes for helical TomoTherapy patients.

**Figure 3:**
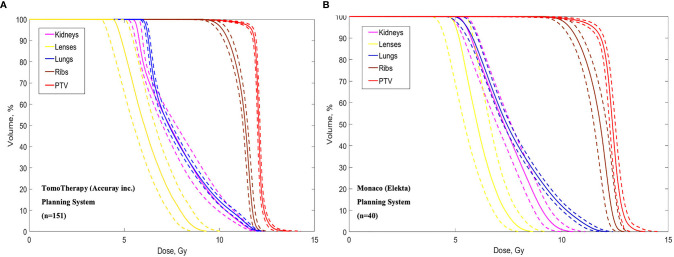
Dose volume histograms calculated in the TomoTherapy **(A)** and Monaco **(B)** Planning systems. Dotted lines show standard deviations.

Average dose volume histograms (DVHs) calculated for the 151 patients treated using TomoTherapy and 40 patients treated using VMAT are shown in **Figure 3**.

All VMAT-based plans were created using multi-isocenters technique. The treatment isocenters are set up on separate parts of the PTV_Body (head, chest, abdomen, pelvis). The isocenter number was from 4 to 9 and correlated with patient’s height. For PTV_Legs in Elekta patients, we used two different strategies: for small patients (105–145 cm), we used VMAT technique with two treatment isocenters, and for bigger patients (from 145 cm), we used several IMRT beams with static gantry position and couch rotation to 90°/270°.

**Figure 4:**
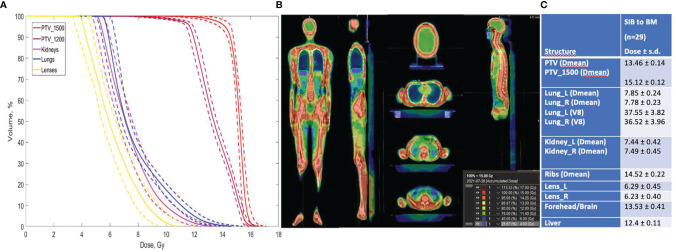
**(A)** Dose volume histograms in the TomoTherapy and Monaco (Elekta) Planning System for pts with SIB to BM (*n* = 29). Dotted lines show standard deviations. **(B)** Treatment plan for TBI + SIB to BM, calculated in TPS TomoTherapy (Accuray Inc.) Planning System. **(C)** Treatment plan calculation results.

Average DVHs for the patients who received SIB to BM (TomoTherapy: 28 patients; Elekta VMAT: one patient) are presented, and the treatment plan with dose distributions for PTV_1200 (TBI) and PTV_1500 (TMI) is shown in **Figure 4**.

## Dosimetric QA

Quality assurance included absolute dosimetry for each treatment plan using dose measurements with an ionization chamber (ExtraDIN IND Chambers, A1SL), 8-chanel electrometer (Tomo Elektrometer from Standart Imaging) and tissue equivalent phantom (Cheese Phantom). For the VMAT-based plans, individual checks included composite measurements of the two-dimensional dose distributions using an array of ionization chambers MatriXX (IBA Dosimetry).

## Radiation Therapy Treatment Characteristics

### Fractionation

**Figure 5:**
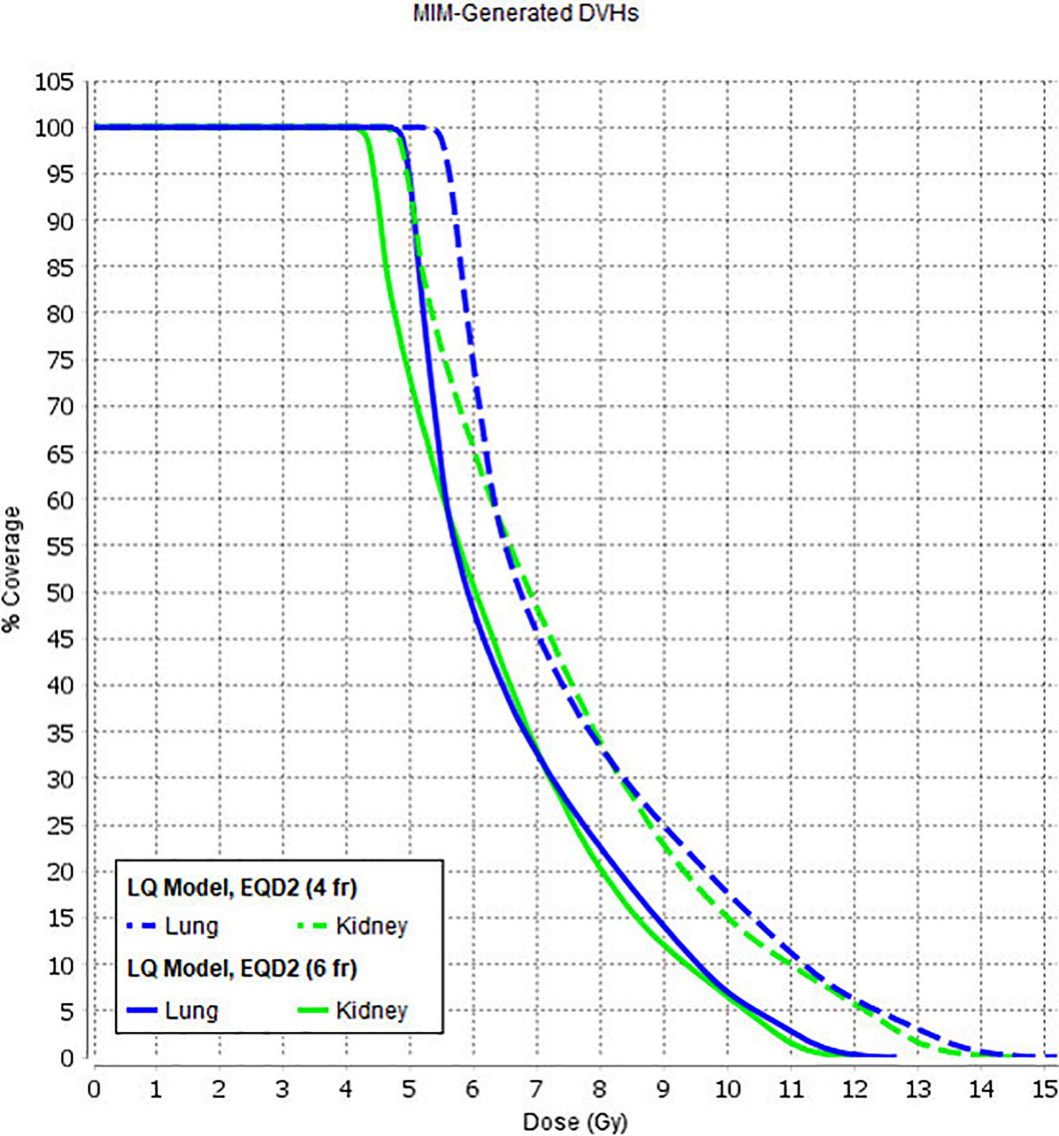
Lung and kidney DVH variations with a linear quadratic model using different alpha/beta values.

The treatment was carried out daily, with the fractions given twice a day with an interfraction interval of 5–6 h over 3 days (group 1). Since April 2020, we have revised the fractionation schedule and reduced the number of treatment sessions to minimize patient/staff contact due to COVID-19. The new treatment schedule included one treatment session per day with a single dose of 3.0 Gy (group 2). Radiobiology modeling supposes that increased dose-per-fraction is associated with higher normal tissue toxicity (36). To determine the effect of this new fractionation schedule, we calculated DVHs using a linear quadratic model using different alpha/beta values with the help of MIM Maestro™ to assess the influence of increased single doses for organs at risk (lungs, kidneys). The increased single dose did not significantly affect the prescribed dose values for the lungs and kidneys (**Figure 5**).

### Treatment Procedure

Positioning was verified prior to each treatment session using megavoltage (MV) CTs and cone-beam (CB)-CTs with subsequent corrections of the setup errors.

The dose delivery time ranged from 16 to 50 min (average 30 min) and was dependent on the patient’s height. Dose delivery times were approximately the same for both the TomoTherapy and VMAT approaches. Total treatment time, including imaging and patient setup, was significantly different for the two methods, with up to 60 min for TomoTherapy and up to 90 min for VMAT.

Radiation therapy treatment was held with a 24-h intravenous infusion (Sodium Chloride 0,9% + sodium bicarbonate—125 ml/h). All the patients received preventive antivomiting prescription (antagonist of the 5-HT3 receptors: 4–8 mg, dexamethasone: 4–6 mg) once per day.

## Statistical Analysis

Statistical analysis was performed using XLSTAT (Addinsoft, 2021) software.

Two hundred and twenty patients who received OC-TBI and underwent allogenic HSCT with TCRαβ/CD-19 depletion were included in the final analysis. Overall survival (OS) and event-free survival (EFS) were calculated using the Kaplan-Meier method. OS was defined as the probability of survival, regardless of disease status, from the time of TBI to the time of death or of the last follow-up (surviving patients were censored at the last follow-up, whereas only death from any cause was considered an event). The following events were considered for EFS: death from any cause, relapse and progression of the main disease (patients with advanced disease). We calculated transplant-related mortality (TRM) and relapse according to the competing risk model, where these two events were considered to be mutually competitive.

## Radiation-Induced Toxicity Evaluation and Results

We followed up patients for acute toxicity (nausea/vomiting/diarrhea, headache, veno-occlusive disease (VOD))—during radiation therapy and 30 days after SCT, subacute toxicity (IP)—up to the 100th day after SCT and late toxicity in the lungs and kidneys—for at least 100 days after SCT in accordance with the RTOG/EORTC scale (37).

### Acute Toxicity: Results

Acute toxicity during radiation therapy was expressed in nausea and vomiting and headache, symptoms of parotitis and enteritis. We observed a correlation between the frequency of nausea/vomiting (*p* = 0.02) with the amount of a single radiation dose. We also noticed the larger number of patients with headache in 3 Gy\fr group, and it seemed to be correlated with the amount of a single dose, but was not statistically significant (*p* > 0.05) (**Table 4**).

**Table 4:** Radiation-induced acute toxicity.

Toxicity criteria (RTOG)	GROUP 1	GROUP 2	*p*-value
	2 Gy × 6 fractions/twice daily	3 Gy × 4 fractions	
Number of pts	201	19	
Nausea and vomit			
• Grades 0–1	124 (62%)	6 (32%)	0.020
• Grades 2–3	77 (38%)	13 (68%)	
Headache			
• Grades 0–1	114 (56%)	12 (63%)	>0.05 (0.751)
• Grades 2–3	87 (44%)	7 (39%)	
Parotitis			
• No clinical symptoms	109 (54%)	9 (47%)	>0.05 (0.755)
• Grade 1 clinical symptoms	92 (46%)	10 (53%)	
Enteritis			
• No clinical symptoms	122 (61%)	10 (53%)	>0.05 (0.733)
• Grade 1	64 (32%)	7 (36%)	
• Grade 2	15 (7%)	2 (11%)	

All patients were able to complete the planned radiation treatment program and received HSCT.

### Subacute Toxicity: Transplant-Related Toxicity and Death

Subacute toxicity among the patients who received OC-TBI (*n* = 191) was observed in 0.4% of the patients (*n* = 1) (interstitial pneumonia, 3–4 stage according to RTOG) at +81 days after TBI (**Table 5**). The patient died from respiratory failure. No radiation-induced kidney toxicity was observed among the patients.

**Table 5:** Radiation-induced subacute toxicity and causes of death.

Patient No.	Ds	TBI	Clinical manifestation	Time of manifestation	Number of HCST	Chemotherapy	Result
1	ALL	12 Gy	IP*	+81 days after TBI	1st (MUD)	Fludarabine 150 mg/m^2^ + thiotepa 10 mg/kg	*Death*
							Respiratory failure
2	ALL	12 Gy + SIB to BM 15 Gy	VOD*	+21 days after TBI	2nd (Haplo)	Fludarabine 150 mg/m^2^ + thiotepa 10 mg/kg	Hepatic failure symptoms
							*Death*
							Relapse
3	AML, M2	12 Gy + SIB to BM 15 Gy	VOD*	+14 days after TBI	2nd (Haplo)	Fludarabine 150 mg/m^2^ + thiotepa 300 mg/kg	*Death*
							Relapse
4	AML, M4	12 Gy + SIB to BM 15 Gy	VOD*	+26 days after TBI	1st (Haplo)	Fludarabine 150 mg/m^2^ + thiotepa 10 mg/kg + Velcade 1.3 mg/m^2^	*Death*
							Relapse

*IP - intersticial pneumonia.

*VOD - venoocclusive disease.

Among the patients who received SIB to bone marrow up to 15 Gy (29 pts), we observed 3 (10%) cases of veno-occlusive disease (VOD), which appeared on days +14, + 21, and +26. One patient developed transitory hepatic failure symptoms (**Table 5**). All these patients died from the disease relapse.

The cumulative incidence of TRM for the entire patient cohort was 10.7 (95% CI: 2.2–16).

Most of the TRM cases were induced by infection and subsequent sepsis with multiorgan failure (*n* = 20). One patient died because of the COVID-19 infection. The full list of infections and its localization is displayed in **Table 6**.

**Table 6:** TRM-related death characteristic (*n* = 22).

TRM-related death	Number of patients (% of all 220 patients)	Cumulative incidence (%)
All patients	**22 (10)**	**10.7 (95% CI, 2.2–16)**
**1st HSCT (180 patients)**	**15 (7)**	**8.7 (95% CI, 5.5–15)**
TRM <100 days	7	3.8 (95% CI, 1.8–8)
TRM >100 days	8	5.0 (95% CI, 2.5–10)
**2nd HSCT (40 patients)**	**7 (3)**	**18.7 (95% CI: 9.5–37.5)**
TRM <100 days	4	10.0 (95% CI, 4.0–25.3)
TRM >100 days	3	8.0 (95% CI: 2.6–24.0)
**TRM <100 days**	**11 (5)**	**5.0 (95% CI: 1.5–8.9)**
CR (181 patients)	7	3.9% (95% CI: 1.4–8.0)
AD patients (39 patients)	4	9.0% (95% CI: 5.0–9.6)
**TRM >100 days**	**11 (5)**	**5.7 (95% CI: 1.7–10.2)**
CR (181 patients)	9	5.8 (95% CI: 1.9–11.0)
AD (39 patients)	2	5.3 (95% CI: 1.4–20.0)
**Cause of death**		
Infection	20 (9)	
Sepsis		
- Bacterial (*Pseudomonas aeruginosa*, *Klebsiella pneumonia*)	9 (4)	
- Fungal and mixed infection (*Aspergillosis*, *Candidiasis*, CMV)	3 (1.3)	
Lung infection		
- Bacterial (*Klebsiella pneumonia*, CMV, ADV)	5 (2)	
- Mixed infection	2 (0.8)	
Gastrointestinal + skin infection		
- Zygomycosis	1 (0.4)	
Interstitial pneumonia (IP)	1 (0.4)	
Other		
COVID-19	1 (0.4)	

CMV, cytomegalovirus; ADV, adenovirus. The bold values defines patients groups sorted by difference criteria (i.e. number of transplantation, TRM time).

**Figure 6:**
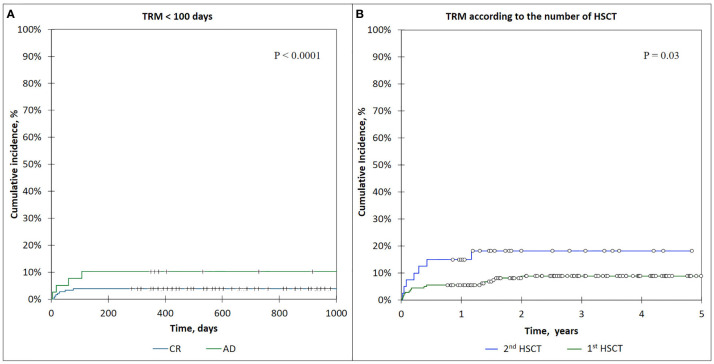
**(A)** The cumulative incidence of TRM <100-day curves, comparison of CR and AD patients. **(B)** The incidence of TRM in patients according to the number of HSCTs.

The incidence of TRM for the patients who received 1st HSCT was 8.7% (95% CI: 5.5–15), and it had significantly higher values for the patients with 2nd HSCT—18.7% (95% CI: 9.5–37.5) (*p* = 0.03) (see **Figure 6**).

The cumulative incidence of early TRM (<100 days) was 5.0 (95% CI: 1.5–8.9), and that of late TRM (>100 days) was 5.7 (95% CI: 1.7–10.2).

The incidence of early TRM (<100 days) was significantly lower for the patients who were in complete remission (CR) before HSCT—3.9% (95% CI: 1.4–8.0) compared with active disease (AD) patients—9.0% (95% CI: 5.0–9.6) (*p* < 0.0001) (**Figure 6**).

### Survival Analysis

We retrospectively analyzed a cohort of 220 patients who received OC-TBI with subsequent allogenic SCT with TCRαβ/CD-19 depletion in our clinic during a period of time from July 2014 to September 2020.

The follow-up period was from 0.3 to 6.4 years (the median follow-up for the surviving patients—2.8 years).

The OS for all patients was 63% (95% CI: 56–70), and the EFS was 58% (95% CI: 51–65).

The OS in the patients with acute leukemia was 63% (95% CI: 43–71) in the ALL group and 52% (95% CI: 32–72) in the AML group (*p* = 0.09). The EFS for the patients with ALL was 57% (95% CI: 49-65), and the EFS for the patients with AML was 52% (95% CI: 32–72) (*p* = 0.3) (**Table 7**).

**Table 7:** The survival analysis results for the different patient groups.

Disease	OS % (95%Cl)	EFS % (95%Cl)	Relapse site (number of pts)
**All patients**	63 (56-70)	58 (51-65)	
ALL	63 (55-71)	57 (49-65)	BM – 38
			BM + CNS – 6
			BM + Testicles – 1
			BM + uterus + ovaries - 1
			BM + bones - 2
			Isolated CNS - 1
			
AML	52 (32-71)	52 (32-72)	BM – 7
			BM + Testicles + EM bones – 1
			BM + CNS – 1
			EM (bones) -1
Other (NHL, Biphenotypic/bilineal leukemia, JMML)	71 (54-88)	70 (53-86)	BM – 3
			BM + EM sites - 3
**Disease status at HSCT**	**OS % (95%Cl)**	**EFS % (95%Cl)**	
CR 1/2/3	68 (60–76)	63 (56-70)	BM – 36
(182 pts)			BM + CNS – 6
			BM + EM sites – 4
			BM + Testicles - 1
			BM + uterus + ovaries – 1
			Isolated CNS - 1
AD (39 pts)	36 (20-52)	36 (20-52)	
AD SIB to BM + (19 pts)	47 (25-70)	47 (25-70)	BM – 4
			BM + EM - 3
AD SIB to BM – (20 pts)	29 (8-49)	27 (6-48)	BM – 8
			BM + CNS – 1
			BM + EM - 2

BM, bone marrow; CNS, central nervous system; EM, extramedullary site.

The OS and EFS for patients with other diseases (NHL, biphenotypic/bilinear leukemia, JMML, etc.) were more related to patients with ALL and AML, with an OS of 71% (95% CI: 54–88) and EFS of 70% (95% CI: 53–86). These results are attributed to the different disease characteristics in this group and are not significant (**Table 7**).

The OS and EFS values in the patients with CR before HSCT (*n* = 181) were 68% (95% CI: 60–76) and 63% (95% CI: 56–70), respectively.

**Figure 7:**
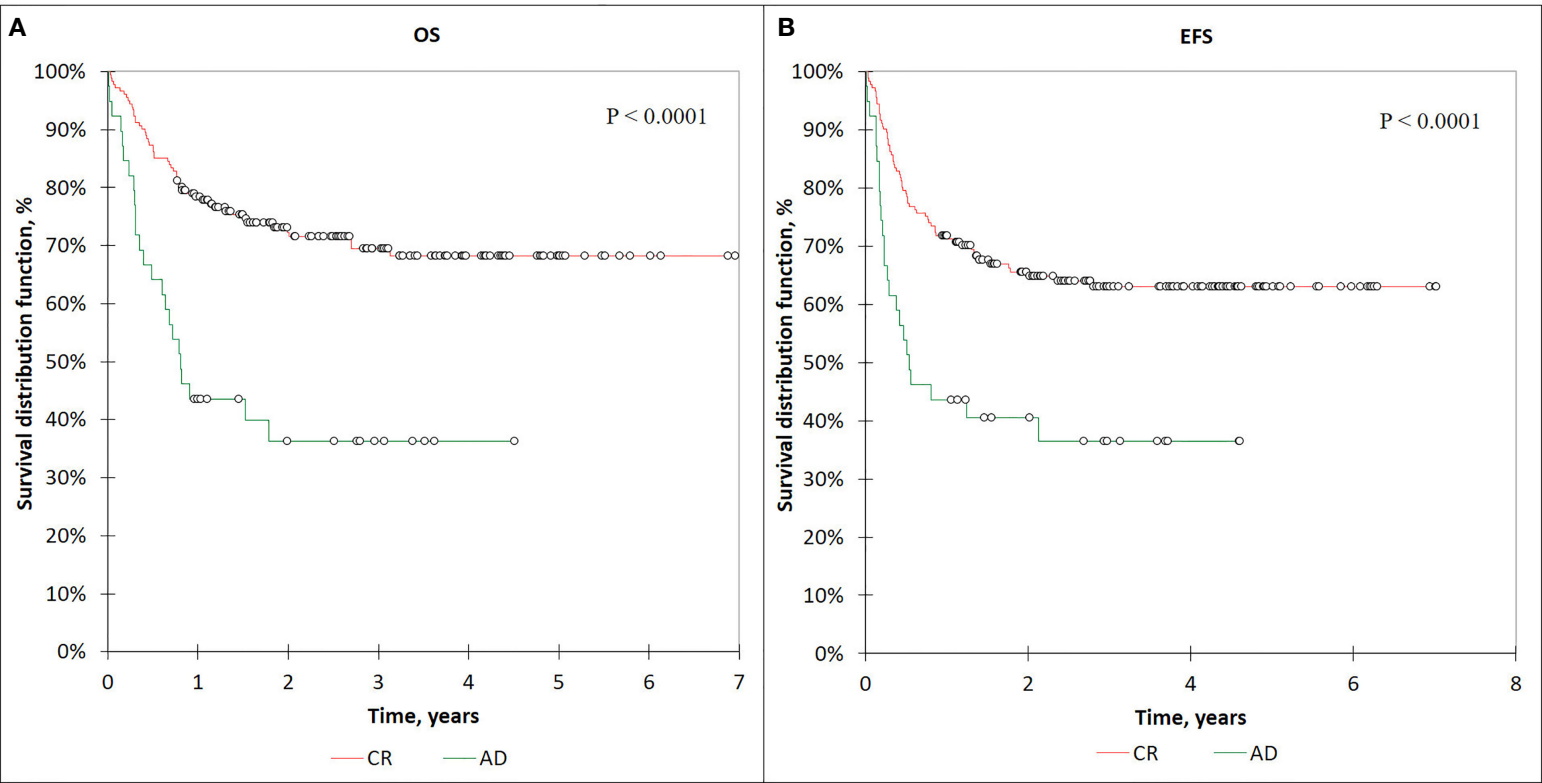
The OS **(A)** and EFS **(B)** curves for the CR and AD patients.

The OS and EFS values for the patients with AD prior to HSCT were significantly lower: OS 36% (95% CI: 20–52) and EFS 36% (95% CI: 20–52) compared with the CR (*n* = 181) patients (*p* < 0.0001) (**Figure 7**). The patients who received treatment in the CR relapsed from the following sites: 36 patients had bone marrow relapse, one patient with ALL had isolated CNS relapse, 12 patients had combined relapses from bone marrow and extramedullary sites (**Table 7**).

### Active Disease Patients

A total of 39 patients had an active disease status prior to HSCT. Among those, 19 patients received conformal TBI with an additional dose escalation (SIB) to BM (Boost+), and 20 patients received 12 Gy conformal TBI (Boost−). We calculated and compared OS, EFS, and cumulative incidence of TRM and relapse in these two groups of patients.

**Figure 8:**
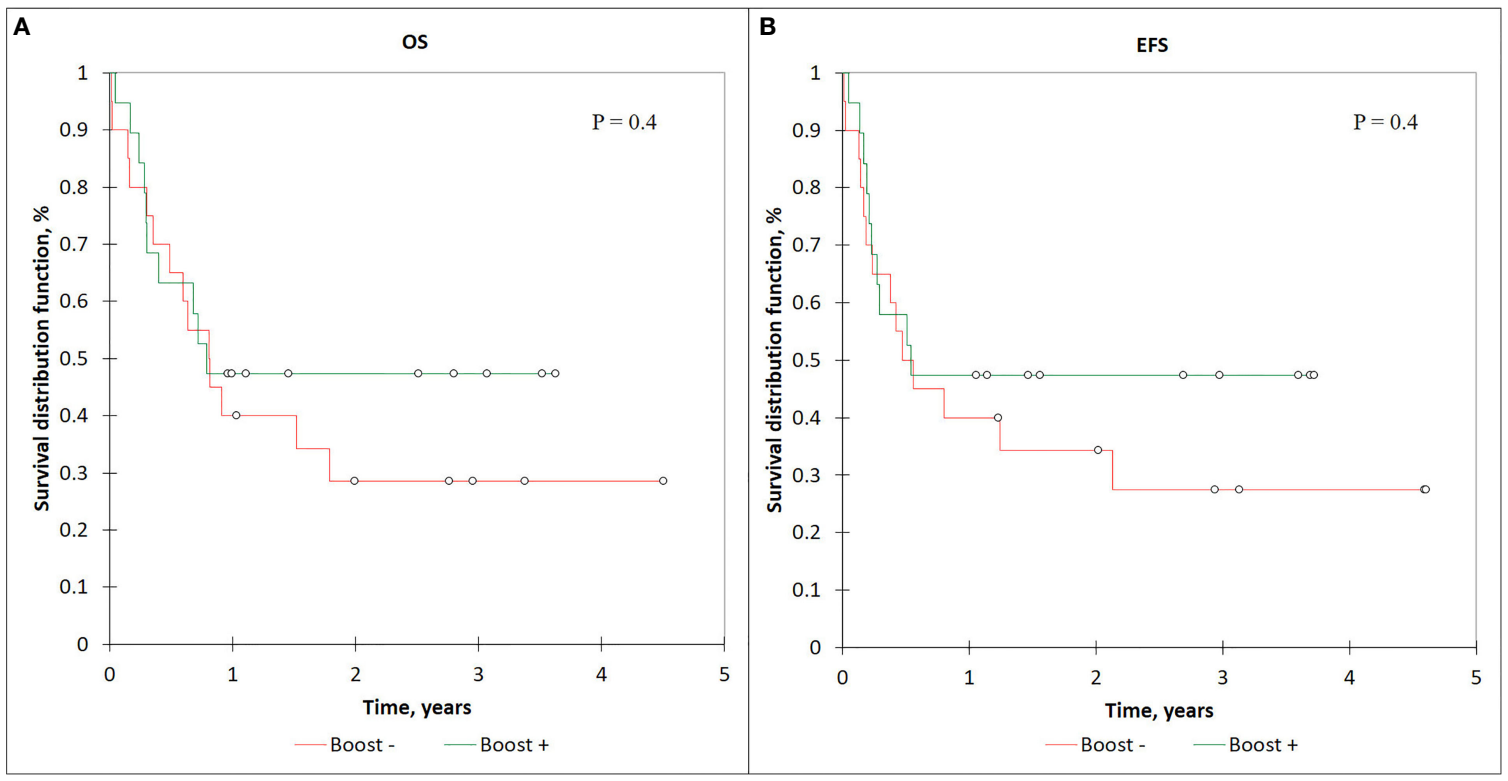
OS **(A)** and EFS **(B)** curves for AD patients who received SIB to BM.

We noticed the difference in the OS and the EFS among this group of patients: the OS and EFS for the Boost+ patients had the same value and was 47% (95% CI: 25–70), while among the Boost− patients OS, and EFS was 29% (95% CI: 8–49) and 27% (95% CI: 6–48) (*p* = 0.4) (**Figure 8** **)**. The lower number of bone marrow recurrence/disease progression cases were registered in the Boost+ patients compared with the Boost− group—five (26%) versus nine (45%). The difference between bone marrow relapse rate in the described groups was not statistically significant (*p*-value = 0.342; Chi-square test). We did not observe CNS relapses in patients receiving SIB to BM. Meanwhile, the cumulative incidence of TRM between the Boost− and Boost+ groups was equal, with a value of 15.8% (95% CI: 8–45).

## Discussion

The main aim of this study was to develop an optimized approach toward TBI planning in pediatric patients using IMRT and TomoTherapy-based conformal avoidance techniques and to evaluate the safety and potential efficacy of this treatment for patients with varying characteristics and disease features.

We did not observe any significant effect of OC-TBI with the described dose limits to the OAR organs at risk on transplant-related mortality. We observed that a single day of fractionation with a higher single dose increased acute radiation-induced toxicity (nausea/vomiting and headache). However, an additional investigation of other nonlethal toxicities for a larger number of patients and a longer follow-up period is required to elucidate disadvantages.

**Figure 9:**
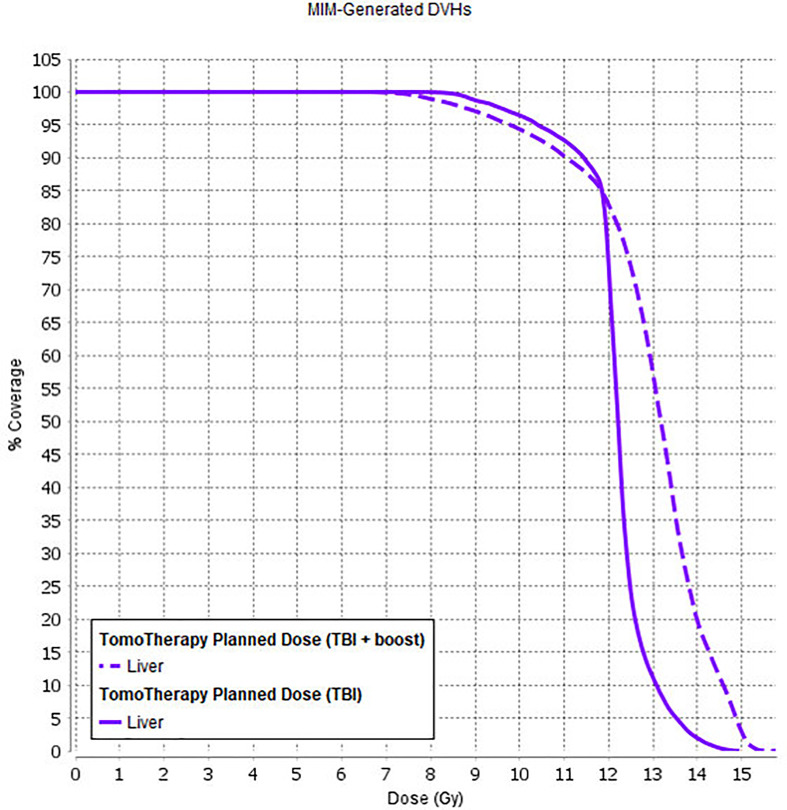
Liver dose for patients who received OC-TBI (solid line) and for patients who received OC-TBI with SIB to BM (dashed line).

Considering the long-term survival period in a pediatric cohort with remission and a good response to received therapy, the development of radiation-induced late side effects (such as hormone dysfunction, hypogonadism, cognitive dysfunction, secondary malignancies) significantly influences the patient’s quality of life. In this manner, a preferred option for the TBI method would be to use it in combination with total bone marrow irradiation (TMI) and/or total lymphoid irradiation (TMLI). This approach would facilitate redistribution of the radiation dose within the patient’s body, leading to dose reduction to the OAR (such as gonads, thyroid, liver, etc.) with a simultaneous increase in the total dose to the bone marrow. Local dose escalation to sanctuary sites (such as bone marrow) with higher doses (up to 18–20 Gy) would likely lead to an increase in the survival rates for active disease patients (**Figure 8**), but it can also be complex in terms of higher toxicity incidence. We explored the initial feasibility of using IMRT and TomoTherapy techniques for planning and delivery of simultaneous additional dose escalation (SIB) to the bone marrow (BM) up to 15 Gy in patients with refractory leukemia. During the early follow-up, we observed a higher incidence of VOD in this group of patients [*n* = 3 (10%)]. The EQD2-calculated liver dose for these patients and its comparison with OC-TBI are presented in **Figure 9**.

Taking into account the published data on the risk factors for VOD development (38–40), we assume that this issue can be resolved by further reducing the dose to the liver parenchyma. Even though TMLI provides a more conformally targeted radiotherapy for patients undergoing HCT, organ sparing could potentially increase the risk of extramedullary relapse and decrease the efficacy of radiation treatment.

We had early experience with seven patients who received a total marrow dose of 15 Gy plus a total lymphoid dose of 12 Gy irradiation using TMLI. At this point, it is too early to draw any conclusions regarding the effectiveness and survival rates for this treatment approach. Nevertheless, taking into account the VOD cases among the patients who received OC-TBI with SIB to bone marrow, we decided to replace this method with total marrow and lymphoid irradiation (with the additional dose reduction to the heart, liver, intestine, thyroid, ovaries, etc.). We saved a 12-Gy treatment dose to the most susceptible extramedullary sites for patients with pediatric leukemia (brain; forehead area for younger patients - **Figure 2**; testicles) and controlled the dose in these areas (D95 >11.4 Gy).

The concept of conformal total body irradiation (TBI) using IMRT VMAT and helical treatment delivery on a TomoTherapy accelerator provides the maximum control of the dose distribution in extended targets (PTV) with a simultaneous dose decrease in organs at risk. It leads to a reduced incidence of severe side effects after radiation therapy and high treatment effectiveness.

OC-TBI with precise dose prescription for organs at risk and image-guided treatment delivery allows us to estimate potential efficacy and radiation-induced toxicity in comparable groups of treated patients. The limited number of pediatric patients who receive OC-TBI requires multidisciplinary and multicenter collaboration and discussion to create new ideas and improve the approach for this complex radiation treatment procedure.

## Data Availability Statement

The original contributions presented in the study are included in the article/supplementary material. Further inquiries can be directed to the corresponding author.

## Ethics Statement

The studies involving human participants were reviewed and approved by Local Ethics Committee of the Dmitriy Rogachev National Medical Research center of pediatric hematology, oncology and immunology. Written informed consent to participate in this study was provided by the participants’ legal guardian/next of kin. Written informed consent was obtained from the individual(s) and minor(s)’ legal guardian/next of kin for the publication of any potentially identifiable images or data included in this article.

## Author Contributions

DA collected and analyzed data and wrote the paper. AN, LS, and MM designed the study, analyzed data and reviewed the paper. FK, DT, and AL collected and analyzed data and wrote the paper. RK and MI collected and analyzed data and reviewed the paper. DB and NM contributed to study design and reviewed the paper. All authors contributed to the article and approved the submitted version.

## Conflict of Interest

The authors declare that the research was conducted in the absence of any commercial or financial relationships that could be construed as a potential conflict of interest.

## Publisher’s Note

All claims expressed in this article are solely those of the authors and do not necessarily represent those of their affiliated organizations, or those of the publisher, the editors and the reviewers. Any product that may be evaluated in this article, or claim that may be made by its manufacturer, is not guaranteed or endorsed by the publisher.

## References

[1] Davies SM Ramsay NK Klein JP Weisdorf DJ Bolwell B Cahn JY. Comparison of Preparative Regimens in Transplants for Children With Acute Lymphoblastic Leukemia. J Clin Oncol (2000) 18(2):340–7. doi: 10.1200/jco.2000.18.2.340 10637248

[2] Blume KG Forman SJ Snyder DS Nademanee AP O’Donnell MR Fahey JL. Allogeneic Bone Marrow Transplantation for Acute Lymphoblastic Leukemia During First Complete Remission. Transplantation (1987) 43(3):389–92. doi: 10.1097/00007890-198703000-00014 3547796

[3] Glasgow GP Wang S Stanton J. A Total Body Irradiation Stand for Bone Marrow Transplant Patients. Int J Radiat Oncol Biol Phys (1989) 16(3):875–7. doi: 10.1016/0360-3016(89)90508-7 2646267

[4] Mody R Li S Dover DC Sallan S Leisenring W Oeffinger KC. Twenty-Five–Year Follow-Up Among Survivors of Childhood Acute Lymphoblastic Leukemia: A Report From the Childhood Cancer Survivor Study. Blood (2008) 111(12):5515–23. doi: 10.1182/blood-2007-10-117150 PMC2424150 18334672

[5] Ozsahin M Pène F Touboul E Gindrey-Vie B Dominique C Lefkopoulos D. Total-Body Irradiation Before Bone Marrow Transplantation. Results of Two Randomized Instantaneous Dose Rates in 157 Patients. Cancer (1992) 69(11):2853–65. doi: 10.1002/1097-0142(19920601)69:11<2853::AID-CNCR2820691135>3.0.CO;2-2 1571917

[6] Locatelli F Merli P Pagliara D Li Pira G Falco M Pende D. Outcome of Children With Acute Leukemia Given HLA-Haploidentical HSCT After αβ T-Cell and B-Cell Depletion. Blood (2017) 130(5):677–85. doi: 10.1182/blood-2017-04-779769 28588018

[7] Blaise D Maraninchi D Michallet M Reiffers J Jouet JP Milpied N. Long-Term Follow-Up of a Randomized Trial Comparing the Combination of Cyclophosphamide With Total Body Irradiation or Busulfan as Conditioning Regimen for Patients Receiving HLA-Identical Marrow Grafts for Acute Myeloblastic Leukemia in First Complete Remission. Blood (2001) 97(11):3669–71. doi: 10.1182/blood.V97.11.3669 11392326

[8] Kelsey CR Horwitz ME Chino JP Craciunescu O Steffey B Folz RJ. Severe Pulmonary Toxicity After Myeloablative Conditioning Using Total Body Irradiation: An Assessment of Risk Factors. Int J Radiat Oncol Biol Phys (2011) 81(3):812–8. doi: 10.1016/j.ijrobp.2010.06.058 20932682

[9] Abugideiri M Nanda RH Butker C Zhang C Kim S Chiang KY. Factors Influencing Pulmonary Toxicity in Children Undergoing Allogeneic Hematopoietic Stem Cell Transplantation in the Setting of Total Body Irradiation-Based Myeloablative Conditioning. Int J Radiat Oncol Biol Phys (2016) 94(2):349–59. doi: 10.1016/j.ijrobp.2015.10.054 26853343

[10] Hanania AN Mainwaring W Ghebre YT Hanania NA Ludwig M. Radiation-Induced Lung Injury: Assessment and Management. Chest (2019) 156(1):150–62. doi: 10.1016/j.chest.2019.03.033 PMC8097634 30998908

[11] Carruthers SA Wallington MM. Total Body Irradiation and Pneumonitis Risk: A Review of Outcomes. Brit J Cancer (2004) 90(11):2080–84. doi: 10.1038/sj.bjc.6601751 PMC2409505 15150598

[12] Chiang Y Tsai CH Kuo SH Liu CY Yao M Li CC. Reduced Incidence of Interstitial Pneumonitis After Allogeneic Hematopoietic Stem Cell Transplantation Using a Modified Technique of Total Body Irradiation. Sci Rep-UK (2016) 6(1):1–11. doi: 10.1038/srep36730 PMC5103225 27830767

[13] Cosset JM Baume D Pico JL Shank B Girinski T Benhamou E. Single Dose Versus Hyperfractionated Total Body Irradiation Before Allogeneic Bone Marrow Transplantation: A non-Randomized Comparative Study of 54 Patients at the Institut Gustave-Roussy. Radiother Oncol (1989) 15(2):151–60. doi: 10.1016/0167-8140(89)90129-1 2669036

[14] Travis EL Peters LJ McNeill J Thames HD Jr Karolis C. Effect of Dose-Rate on Total Body Irradiation: Lethality and Pathologic Findings. Radiother Oncol (1985) 4(4):341–51. doi: 10.1016/S0167-8140(85)80122-5 3909241

[15] Esiashvili N Lu X Ulin K Laurie F Kessel S Kalapurakal JA. Higher Reported Lung Dose Received During Total Body Irradiation for Allogeneic Hematopoietic Stem Cell Transplantation in Children With Acute Lymphoblastic Leukemia is Associated With Inferior Survival: A Report From the Children’s Oncology Group. Int J Radiat Oncol Biol Phys (2019) 104(3):513–21. doi: 10.1016/j.ijrobp.2019.02.034 PMC6548591 30807822

[16] Sampath S Schultheiss T Wong J. Dose Response and Factors Related to Interstitial Pneumonitis After Bone Marrow Transplant. Int J Radiat Oncol Biol Phys (2005) 63(3):876–84. doi: 10.1016/j.ijrobp.2005.02.032 16199317

[17] Barrett A Nicholls J Gibson B. Late Effects of Total Body Irradiation. Radiother Oncol (1987) 9(2):131–5. doi: 10.1016/S0167-8140(87)80200-1 3303161

[18] Hui SK Kapatoes J Fowler J Henderson D Olivera G Manon RR. Feasibility Study of Helical Tomotherapy for Total Body or Total Marrow Irradiation. Med Phys (2005) 32(10):3214–24. doi: 10.1118/1.2044428 16279075

[19] Wong JY Liu A Schultheiss T Popplewell L Stein A Rosenthal J. Targeted Total Marrow Irradiation Using Three-Dimensional Image-Guided Tomographic Intensity-Modulated Radiation Therapy: An Alternative to Standard Total Body Irradiation. Biol Blood Marrow Tr (2006) 12(3):306–15. doi: 10.1016/j.bbmt.2005.10.026 16503500

[20] Schultheiss TE Wong J Liu A Olivera G Somlo G. Image-Guided Total Marrow and Total Lymphatic Irradiation Using Helical Tomotherapy. Int J Radiat Oncol Biol Phys (2007) 67(4):1259–67. doi: 10.1016/j.ijrobp.2006.10.047 17336225

[21] Wong JY Rosenthal J Liu A Schultheiss T Forman S Somlo G. Image-Guided Total-Marrow Irradiation Using Helical Tomotherapy in Patients With Multiple Myeloma and Acute Leukemia Undergoing Hematopoietic Cell Transplantation. Int J Radiat Oncol Biol Phys (2009) 73:273–9. doi: 10.1016/j.ijrobp.2008.04.071 PMC3896447 18786784

[22] Peñagarícano JA Chao M Van Rhee F Moros EG Corry PM Ratanatharathorn V. Clinical Feasibility of TBI With Helical Tomotherapy. Bone Marrow Transpl (2011) 46:929–35. doi: 10.1038/bmt.2010.237 20935684

[23] Gruen A Ebell W Wlodarczyk W Neumann O Kuehl JS Stromberger C. Total Body Irradiation (TBI) Using Helical Tomotherapy in Children and Young Adults Undergoing Stem Cell Transplantation. Radiat Oncol (2013) 8(1):1–8. doi: 10.1186/1748-717X-8-92 23587349 PMC3653702

[24] Konishi T Ogawa H Najima Y Hashimoto S Wada A Adachi H. Safety of Total Body Irradiation Using Intensity-Modulated Radiation Therapy by Helical Tomotherapy in Allogeneic Hematopoietic Stem Cell Transplantation: A Prospective Pilot Study. J Radiat Res (2020) 61(6):969–76. doi: 10.1093/jrr/rraa078 PMC7674702 32888029

[25] Shueng PW Lin SC Chong NS Lee HY Tien HJ Wu LJ. Total Marrow Irradiation With Helical Tomotherapy for Bone Marrow Transplantation of Multiple Myeloma: First Experience in Asia. Tech Canc Res Treat (2009) 8(1):29–37. doi: 10.1177/153303460900800105 19166240

[26] Maschan M Shelikhova L Shekhovtsova Z Balashov D Kurnikova E Muzalevsky Y. TCR Alpha/Beta and CD19 Depletion in Transplantation From Matched Unrelated and Haploidentical Donors in Pediatric Leukemia Patients: Comparison of Two Gvhd Prophylaxis Regimens. Biol Blood Marrow Transplant (2016) 22(3):385–5. doi: 10.1016/j.bbmt.2015.11.901

[27] Maschan M Shelikhova L Ilushina M Kurnikova E Boyakova E Balashov D. TCR-Alpha/Beta and CD19 Depletion and Treosulfan-Based Conditioning Regimen in Unrelated and Haploidentical Transplantation in Children With Acute Myeloid Leukemia. Bone Marrow Transpl (2016) 51(5):668–74. doi: 10.1038/bmt.2015.343 26808573

[28] Shelikhova L Shekhovtsova Z Balashov D Boyakova E Muzalevskyi I Gutovskaya E. Tcrαβ+/CD19+-Depletion in Hematopoietic Stem Cells Transplantation From Matched Unrelated and Haploidentical Donors Following Treosulfan or TBI-Based Conditioning in Pediatric Acute Lymphoblastic Leukemia Patients. Blood (2016) 128(22):4672. doi: 10.1182/blood.V128.22.4672.4672

[29] Loginova AA Kobyzeva DA Tovmasyan DA Chernyaev AP Lisovskaya АО Maschan MA. Comparison of Total Body Irradiation Using TomoTherapy and Volume-Modulated Rotational Radiation Therapy Elekta. A Single Center Experience on Pediatric Patients. Pediatr Hemat/Oncol Immunopath (2019) 18(4):49–57. doi: 10.24287/1726-1708-2019-18-4-49-57

[30] Jiang Z Jia J Yue C Pang Y Liu Z Ouyang L. Haploidentical Hematopoietic SCT Using Helical Tomotherapy for Total-Body Irradiation and Targeted Dose Boost in Patients With High-Risk/Refractory Acute Lymphoblastic Leukemia. Bone Marrow Transpl (2018) 53(4):438–48. doi: 10.1038/s41409-017-0049-5 29330392

[31] Corvò R Zeverino M Vagge S Agostinelli S Barra S Taccini G. Helical Tomotherapy Targeting Total Bone Marrow After Total Body Irradiation for Patients With Relapsed Acute Leukemia Undergoing an Allogeneic Stem Cell Transplant. Radiat Oncol (2011) 98(3):382–6. doi: 10.1016/j.radonc.2011.01.016 21339008

[32] Borg M Hughes T Horvath N Rice M Thomas AC. Renal Toxicity After Total Body Irradiation. Int J Radiat Oncol Biol Phys (2002) 54(4):1165–73. doi: 10.1016/S0360-3016(02)03039-0 12419445

[33] Esiashvili N Chiang KY Hasselle MD Bryant C Riffenburgh RH Paulino AC. Renal Toxicity in Children Undergoing Total Body Irradiation for Bone Marrow Transplant. Radiat Oncol (2009) 90(2):242–6. doi: 10.1016/j.radonc.2008.09.017 18973960

[34] Cheng JC Schultheiss TE Wong JY. Impact of Drug Therapy, Radiation Dose, and Dose Rate on Renal Toxicity Following Bone Marrow Transplantation. Int J Radiat Oncol Biol Phys (2008) 71(5):1436–43. doi: 10.1016/j.ijrobp.2007.12.009 18355974

[35] Chou RH Wong GB Kramer JH Wara DW Matthay KK Crittenden MR. Toxicities of Total-Body Irradiation for Pediatric Bone Marrow Transplantation. Int J Radiat Oncol Biol Phys (1996) 34(4):843–51. doi: 10.1016/0360-3016(95)02178-7 8598361

[36] Wheldon TE. The Radiobiological Basis of Total Body Irradiation. Brit J Radiol (1997) 70(840):1204–7. doi: 10.1259/bjr.70.840.9505837 9505837

[37] Cox JD Stetz J Pajak TF. Toxicity Criteria of the Radiation Therapy Oncology Group (RTOG) and the European Organization for Research and Treatment of Cancer (EORTC). Int J Radiat Oncol Biol Phys (1995) 31(5):1341–46. doi: 10.1016/0360-3016(95)00060-C 7713792

[38] Mohty M Malard F Abecassis M Aerts E Alaskar AS Aljurf M. Sinusoidal Obstruction Syndrome/Veno-Occlusive Disease: Current Situation and Perspectives—A Position Statement From the European Society for Blood and Marrow Transplantation (EBMT). Bone Marrow Transpl (2015) 50(6):781–9. doi: 10.1038/bmt.2015.52 PMC4456788 25798682

[39] Mcdonald GB Sharma P Matthews DE Shulman HM Thomas ED. Venocclusive Disease of the Liver After Bone Marrow Transplantation: Diagnosis, Incidence, and Predisposing Factors. Hepatology (1984) 4(1):116–22. doi: 10.1002/hep.1840040121 6363247

[40] Coppell JA Richardson PG Soiffer R Martin PL Kernan NA Chen A. Hepatic Veno-Occlusive Disease Following Stem Cell Transplantation: Incidence, Clinical Course, and Outcome. Biol Blood Marrow Transplant (2010) 16(2):157–68. doi: 10.1016/j.bbmt.2009.08.024 PMC3018714 19766729

